# Supine CT-Guided Posterior Approach Using a Dorsal-Space Mat: Initial Clinical Experience and Geometric Characterization

**DOI:** 10.3390/tomography12090134

**Published:** 2026-09-15

**Authors:** Yuya Koike, Tatsuo Ueda, Fumie Sugihara, Masashi Tanaka

**Affiliations:** 1 Department of Cardiovascular Surgery, Nihon University School of Medicine, 30-1, Oyaguchi Kami-cho, Itabashi-ku, Tokyo 173-8610, Japan; tanaka.masashi@nihon-u.ac.jp; 2 Department of Radiology, Nippon Medical School, 1-1-5, Sendagi, Bunkyo-ku, Tokyo 113-8603, Japan; s9015@nms.ac.jp (T.U.); giorcubgogo@nms.ac.jp (F.S.)

**Keywords:** CT-guided procedures, biopsy, radiofrequency ablation, drainage, posterior approach

## Abstract

Background/Objectives: To evaluate the clinical feasibility, safety, and geometric characteristics of a supine CT-guided posterior approach using a custom dorsal-space mat in patients for whom the supine position was required or preferred. Methods: This retrospective study included 16 consecutive patients prepared for the approach. Puncture trajectory parameters, positional stability, technical success, procedure time, and adverse events were evaluated in successfully positioned patients (*n* = 15; 35 trajectories). A geometric model was constructed to estimate maximum allowable puncture angles determined by mat height, needle length, and table geometry. Results: Positioning feasibility was 15/16 (93.8%); one patient was converted to a lateral approach due to shoulder pain. Technical success was 100% per patient (15/15) and per trajectory (35/35). The mean puncture angle and depth were 40.2° ± 9.1° and 8.3 ± 2.5 cm, respectively. All puncture angles were within theoretical geometric limits, with a mean angular margin of 14.9° ± 10.9°. Quantitative stability assessment was feasible in 14 patients; median two-dimensional displacement and absolute axial rotation were 4.1 mm (IQR, 3.3–4.5 mm) and 0.9° (IQR, 0.3–1.2°), respectively. Between-group differences observed in the trajectory-level analysis were not statistically significant in the patient-level sensitivity analysis. One Grade 5 adverse event occurred after RFA; although a relationship with the procedure could not be excluded, it was unrelated to the mat or puncture trajectory. Conclusions: This preliminary feasibility study suggests that the dorsal-space mat can provide sufficient working space for CT-guided posterior procedures while maintaining the supine position. Larger prospective studies are required to establish its broader clinical applicability and safety.

## Simple Summary

Some patients find it difficult to assume the prone position during CT-guided procedures due to pain, obesity, or clinical conditions. To address this, we evaluated a supine posterior approach using a custom dorsal-space mat across various anatomical targets and procedure types. Positioning feasibility was 15/16 (93.8%), and technical success was achieved in all 15 successfully positioned patients (15/15). All 35 puncture trajectories fell within theoretical limits predicted by our geometric model. These preliminary findings suggest that the dorsal-space mat can provide working space for CT-guided interventions in patients for whom the supine position was required or preferred.

## 1. Introduction

CT-guided procedures such as biopsy, drainage, and radiofrequency ablation (RFA) often require a posterior approach when target lesions are located posteriorly, which typically necessitates prone positioning [1,2,3,4,5,6,7,8]. However, in clinical practice, prone or lateral positioning may be difficult or suboptimal in patients with severe pain, respiratory insufficiency, obesity, or those undergoing mechanical ventilation under conscious sedation, making it necessary to maintain the supine position [7,9,10,11]. Several axial approaches along the body axis have been described to access posterior targets while preserving the supine position [9,10,11,12], but these techniques are applicable only in selected cases and generally require advanced operator expertise.

To provide a more universal and reproducible solution, a dedicated dorsal-space mat was developed to create a working space for the posterior approach while maintaining the supine position [13]. A case report of RFA for renal cell carcinoma demonstrated the technical feasibility of this approach; however, its broader applicability across different procedures and anatomical regions has not been systematically evaluated. Furthermore, the geometric constraints that define the maximum allowable needle angle—determined by mat height, needle length, and the distance from the needle entry point to the CT table edge—have not been quantitatively examined.

The primary aim of this study was to report the initial clinical experience of systematically applying a supine CT-guided posterior approach using a dorsal-space mat in selected patients for whom the supine position was required or preferred. The secondary aim was to construct a geometric model to characterize the maximum feasible needle angle and to compare these theoretical constraints with actual puncture trajectories obtained in clinical practice.

## 2. Methods

### 2.1. Study Design and Setting

This retrospective cohort study was approved by the institutional review board. From September 2024 to March 2026, patients who underwent CT-guided procedures using a dorsal-space mat in the supine position at two institutions were included. The procedures included biopsy, drainage, and radiofrequency ablation (RFA). CT scanners used in this study were a 70-cm gantry Brilliance iCT (Philips, Cleveland, OH, USA), a 78-cm SOMATOM Definition AS (Siemens, Forchheim, Germany), and a 90-cm Aquilion LB (Canon, Otawara, Tochigi, Japan). Procedures performed under cone-beam CT (CBCT) guidance using a flat-panel detector angiography system (Infinix Celeve-I INFX-8000C, Canon) were also included.

### 2.2. Participants

Patients were eligible for the dorsal-space mat–assisted posterior approach in the supine position if they met all of the following criteria:

(i) Inability to tolerate prone or lateral positioning, or clinical difficulty in maintaining stable positioning;
(ii) Inadequate access to the target lesion through conventional ventral or lateral approaches without the dorsal-space mat;
(iii) Operator judgment, based on preprocedural CT, that a posterior approach would be feasible and safe using the dorsal-space mat;
(iv) Ability to maintain a stable supine position on the mat allowing adequate working space.

Clinical Rationale and Assessment of Positioning Feasibility:

In conventional practice, when prone positioning is poorly tolerated, alternative techniques such as semi-prone positioning with chest/pelvic wedges or supported lateral decubitus positioning are often considered. However, in our clinical experience, these partial positioning techniques frequently suffer from intraprocedural positional instability or subtle body shifts, which can alter the puncture trajectory planned on preprocedural supine CT images or cause loss of a safe puncture window. Therefore, in this preliminary feasibility study, the dorsal-space mat–assisted supine posterior approach was actively selected not only for patients who were completely unable to tolerate prone or lateral positioning (due to pain, obesity), but also for patients in whom remaining supine was judged by the operator to provide a markedly safer, more reproducible, and more stable puncture trajectory than attempting unstable partial prone or lateral positioning. Intolerance or instability during prone/lateral positioning was determined by the attending interventional radiologist through preprocedural patient interviews or trials on the CT table.

Exclusion Criteria and Endpoints:

Patients unable to maintain a stable supine position or achieve an adequate working trajectory on the dorsal-space mat were classified as cases of difficult positioning and were excluded from the assessment of technical success, procedural success, complications, and trajectory analysis. Positioning feasibility was defined as the proportion of patients who successfully maintained stable positioning with adequate working space on the mat to proceed with the planned posterior puncture. Technical success was evaluated both per patient and per trajectory.

### 2.3. Dorsal-Space Mat and Positioning Technique

A dorsal-space mat was developed, as previously described [13], to maintain the supine position while providing a posterior working space. Because no commercial product was available, the mats were custom-manufactured from polyolefin foam (Marusuzu, Tokyo, Japan). Each mat was 22 cm wide and available in three lengths (180 cm, 90 cm, and 60 cm). Two height options were used: 15 cm at the first institution and 20 cm at the second institution. At the first institution, scanners with a 70-cm gantry diameter could not safely accommodate a 20-cm mat without risking contact between the patient’s upper extremities and the gantry; therefore, a 15-cm mat was selected. At the second institution, scanners with 78-cm and 90-cm gantries allowed safe placement of a 20-cm mat.

Mat length was selected to provide approximately 30 cm of dorsal working space while maintaining a stable supine position. Stability was ensured as long as the shoulders and pelvis were supported, and the mats could be easily repositioned according to the anatomical target. The non-target side of the torso rested on the 180-cm mat, whereas the target side was supported by a 60-cm mat placed beneath the head and shoulders and a 90-cm mat placed beneath the pelvis and lower extremities. The mat was positioned beneath the patient before CT acquisition, and the puncture trajectory was planned using supine CT images obtained with the mat in place.

To prevent patient motion or falls, safety belts were applied around the shoulders and pelvis. The foam mats were firm and exhibited minimal compression under load, providing a stable dorsal working space for posterior interventions.

### 2.4. Intervention

All procedures were performed by board-certified interventional radiologists with over 20 years of experience. All interventions were conducted under local anesthesia, and fentanyl was administered for analgesia during RFA. After planning CT, the puncture site was determined, followed by sterile preparation and draping.

Under CT or CBCT guidance, the needle was advanced toward the target lesion. Needle length was selected primarily according to the skin-to-lesion distance measured on planning CT, although the availability of specific needle lengths occasionally influenced the final choice. In contrast, the puncture trajectory and puncture angle were determined by the operator according to lesion location, surrounding anatomy, and the working space created by the selected mat height. During upward puncture, the operator typically adopted a semi-sitting or crouched posture to visualize the entry point. After advancing the needle through the skin and fascia, plastic forceps were occasionally used to stabilize the needle during image acquisition, preventing deviation and keeping the operator’s hand outside the imaging field for radiation safety.

**Figure 1:**
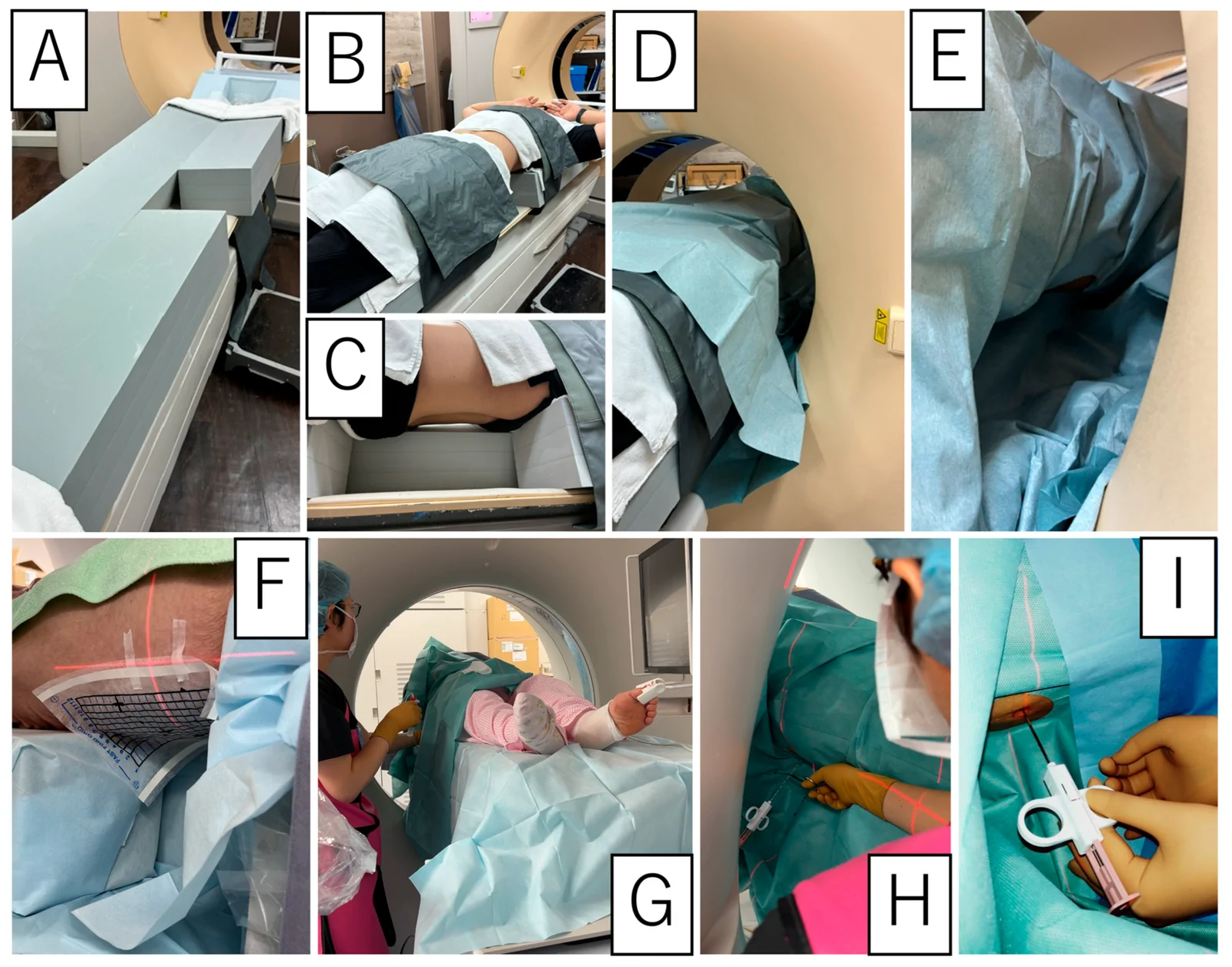
Patient positioning, dorsal space setup, and ergonomics of the upward approach. (**A**) Layout of foam mats on the CT couch: Three hard foam mats are arranged on the CT table to create the dorsal working space. The position and extent of the dorsal space can be adjusted by altering the distance between the mats. (**B**) Volunteer positioning and safety restraint: A healthy volunteer positioned on the mats. Routine safety belts attached to the CT couch are secured across the patient. Because the head, shoulders, buttocks, and lower extremities are supported by the two mats, the patient’s posture remains stable despite the unsupported dorsal space. (**C**) Close-up of the created dorsal space: The setup provides a working space measuring 30–45 cm in width, 15 cm in height (using a 15 cm mat), and 22 cm in depth. (**D**) Positioning within the CT gantry: The draped subject inside the CT gantry. The maximum elevation height is restricted by the gantry aperture. Care is taken to prevent contact between the gantry and the patient’s face or upper limbs during table movement. (**E**) Ergonomic operator hand placement: Close-up of the draped dorsal space demonstrating hand positioning. Resting the operator’s hand/forearm directly on the CT couch provides a stable pivot and minimizes hand fatigue during upward insertion. (**F**) Skin marking in a clinical procedure: Laser marking for entry point localization in an actual patient. When the entry point approaches the dorsal midline, visibility of the red laser reference line decreases due to shadow effects. (**G**) Real-time puncture scene: Overall clinical view during needle placement using the dorsal space. (**H**) Needle stabilization with plastic forceps: Close-up of the hands. At shallow insertion depths, non-magnetic forceps are used to support the needle hub and prevent displacement or dislodgement during verification CT scans. (**I**) Upward trajectory control: Close-up showing the upward angle of needle advancement managed from underneath.

As practical guidance for reproducing the technique, clinical photographs demonstrating the actual procedural setup were provided (Figure 1).

During CBCT-guided puncture under fluoroscopy, the operator’s hand was close to the beam. To minimize radiation exposure, the needle was held with forceps and advanced under intermittent fluoroscopy until sufficient depth was achieved, followed by rotational acquisition.

For biopsy, a 17- or 19-gauge coaxial introducer needle was used, followed by an 18- or 20-gauge biopsy needle (SuperCore, Argon Medical Devices, Frisco, TX, USA; with a total needle length of approximately 17 cm or 23 cm). For drainage, a Drainaway^®^ catheter (SB Kawasumi, Tokyo, Japan; with a total needle length of approximately 22 cm) was placed using the modified trocar technique [14]. For RFA, internally cooled electrodes with a 3-cm active tip (Cool-tip, Covidien, Mansfield, MA, USA; with a total needle length of approximately 20 cm) or a variable VIVARF needle (STARmed, Goyang, Republic of Korea; with a total needle length of approximately 20 cm) were used, ensuring a 5-mm ablation margin.

### 2.5. Variables

Patient characteristics included age, sex, body mass index (BMI), procedure type, and reason for supine restriction. Lesion characteristics included long- and short-axis diameters and skin-to-lesion depth on preprocedural CT. Procedure-related variables included the number of puncture trajectories, needle total length, CT scanner used, and procedure time. Positional stability on the dorsal-space mat was quantitatively assessed by comparing the planning and confirmation CT images at the same vertebral level. The center of the spinal canal was used as a reproducible anatomical landmark, and the medial upper edge of the dorsal-space mat was used as an external reference landmark. The horizontal and vertical distances between these landmarks were measured on both scans. The changes in the horizontal and vertical distances between the two scans were defined as Δ*x* and Δ*y*, respectively, and their absolute values were used to quantify displacement in each direction. Overall two-dimensional displacement was calculated as √(Δ*x*^2^ + Δ*y*^2^). Axial rotation was assessed by measuring the angle of the line connecting the center of the spinal canal and the tip of the spinous process relative to the vertical image axis, and the absolute change in this angle between the two scans was calculated.

Using intraprocedural CT/CBCT images, trajectory parameters were measured, including horizontal skin entry position (*x*_0_), vertical skin entry position (*y*_0_, or entry height), puncture angle, and puncture depth. A two-dimensional Cartesian reference system was defined on axial images, with the vertical axis (*x* = 0) passing through the dorsal midline and the horizontal reference line (*y* = 0) passing through the most posterior point of the body contour. Because the patient and dorsal-space mat could be tilted relative to the CT image plane, the most posterior point of the body contour did not necessarily coincide with the dorsal midline. Accordingly, the coordinate origin (0, 0), defined as the intersection of these two reference lines, did not necessarily correspond to a point on the skin surface. The horizontal skin entry position (*x*_0_) was defined as the horizontal distance from the dorsal midline, and the vertical skin entry position (*y*_0_) as the vertical distance from the horizontal reference line to the actual skin entry point.

Trajectory measurements represented the results of procedures that had already been judged feasible based on preprocedural assessment. Therefore, the measured angles and depths reflect only the characteristics of successfully completed puncture trajectories and do not directly evaluate feasibility in patients with more severe positional limitations.

Technical success was defined as successful needle placement into the target lesion. Procedural success was defined as obtaining diagnostic pathology in biopsy cases, absence of tumor enhancement on discharge CT after RFA, or effective drainage in abscess cases. Adverse events (AEs) were classified as intra-procedural or post-procedural and graded from 1 to 5 according to the Society of Interventional Radiology (SIR) classification [15].

### 2.6. Geometric Model for Maximum Allowable Puncture Angle

Advancing a puncture needle within the space provided by the dorsal-space mat is geometrically constrained by mat height (*H*), total needle length (*L*), CT table width (*W*), and the patient’s dorsal skin contour. To define the theoretical maximum allowable puncture angle permitted by these geometric factors, a theoretical model was constructed. This model was not intended to directly evaluate procedural feasibility in clinical practice, but rather to characterize the upper boundary imposed by table and needle geometry. A two-dimensional Cartesian coordinate system was established using the same coordinate orientation defined for the trajectory measurements: the vertical axis (*x* = 0) passed through the dorsal midline, and the horizontal reference line (*y* = 0) passed through the most posterior point of the body contour. For geometric simplification, the dorsal body surface was assumed to be flat and to lie at the level of the upper surface of the supporting mat. Accordingly, the horizontal reference line (y = 0) in the theoretical model corresponds to the nominal upper-surface level of the mat. In this coordinate frame, the region beneath the patient on the non-target side (−*x*, −*y*) is occupied by the supporting mat, whereas the space beneath the target side (+*x*, −*y*) represents the open working space created by the dorsal-space mat. Target lesions are located internally within the body quadrant (+*x*, +*y*). Fixed geometric parameters included mat height (*H* = 15 or 20 cm), needle length (*L* = 17 or 23 cm), and CT table width (*W* = 40 cm, giving a distance of *W*/2 = 20 cm from the table center to the edge). In this theoretical formulation, the nominal mat height above the table (*H*_table_) was set equal to *H*, which is distinct from the measured entry height (*y*_0_) defined above. The model evaluates the precise moment immediately prior to needle insertion, when the entire needle shaft remains outside the body and its external length equals the total needle length (*L*). Regarding spatial orientation at this moment, the entire needle extends from the external hub, positioned laterally and downward relative to the skin entry point, to the tip at the skin entry point (*x*_0_, *y*_0_), and is directed toward the internal target located in the (+*x*, +*y*) quadrant. Thus, the direction of needle advancement is medial (−*x* direction) and upward (+*y* direction). The maximum allowable puncture angle (*θ*_allow_) was defined as the geometric boundary reached just before either the needle hub or any portion of the needle shaft contacts the CT table surface or table edge at the start of puncture. This model assumes rigid needle behavior, a flat and non-deformable table surface, no compression of the mat or soft tissues, and a flat dorsal body surface extending uniformly to 20 cm lateral to the midline without curvature, spinal deformity, or soft-tissue contour variations.

For each combination of *H* and *L*, the geometric limit imposed by the mat and needle was calculated as follows:

*θ*_needle_ = arcsin(*H*/*L*), for *H* < *L*, and defined as 90° for *H* ≥ *L*.

The angle required to avoid contact with the table edge at puncture position *x*_0_ was calculated as follows:

*θ*_table_ = arctan(*H*_table_/(*W*/2 − *x*_0_)) for *x*_0_ < *W*/2, and defined as 90° for *x*_0_ ≥ *W*/2.

The maximum allowable puncture angle at each position was defined as follows:

*θ*_allow_ = max(*θ*_needle_, *θ*_table_)

Thus, *θ*_allow_ represents the theoretical upper limit beyond which either the needle hub or any portion of the needle shaft physically contacts the CT table structure. Near the dorsal midline (small *x*_0_), *θ*_allow_ is governed by *θ*_needle_, because attempting a steeper angle causes the needle hub to strike the flat table surface before reaching the table edge. As the entry point shifts laterally (larger *x*_0_), the needle clears the table edge (*W*/2 = 20 cm) into open space, releasing the constraint imposed by the table surface and allowing steeper angles governed by *θ*_table_.

### 2.7. Bias

Potential sources of bias included selection bias due to operator-dependent indication for mat use, measurement bias related to intraprocedural CT/CBCT-based angle assessment, and confounding arising from differences in CT equipment between institutions.

### 2.8. Sample and Study Size

The final analysis included 15 patients and 35 puncture tracts. Although multiple tracts within the same patient do not represent independent observations, each tract was analyzed individually when it constituted a distinct puncture route. In biopsy cases, separate tracts were analyzed when samples were obtained from different target sites. In RFA cases, multiple tracts were analyzed when required by lesion size or morphology. Because all eligible cases during the study period were consecutively included, no formal a priori sample size calculation was performed.

### 2.9. Puncture Trajectory Measurement

All geometric trajectory variables, including puncture position (*x*_0_, *y*_0_), puncture angle (*θ*) and depth, were measured on axial CT images using the standard electronic calipers and angle-measurement tools available in the standard clinical image viewer integrated into the electronic medical record system. All measurements followed a unified measurement protocol based on the skin entry point and the needle trajectory visualized on axial slices. Measurements were performed retrospectively by the operators who conducted the procedures (Y.K. and F.S.). Because the measurements were based on predefined anatomical and geometric landmarks and standardized electronic measurement tools, no additional clinical judgment was required for measurement.

### 2.10. Statistical Analysis

Statistical analyses were performed using R version 4.6.1 (R Foundation for Statistical Computing, Vienna, Austria) within RStudio version 2026.06.0 (Posit Software, PBC, Boston, MA, USA). Continuous variables were evaluated for normality using the Shapiro–Wilk test. Normally distributed variables are expressed as mean ± standard deviation (SD) and compared between groups using Welch’s *t*-test, whereas non-normally distributed variables are expressed as median [interquartile range, IQR] and compared using the Wilcoxon rank-sum test. Selected descriptive variables are presented as median (range: min–max). Quantitative positional stability parameters (*n* = 14) were summarized as median [IQR] because of the small sample size and non-normal distributions of some displacement measures. A *p*-value < 0.05 was considered statistically significant.

Puncture trajectories were visualized using scatter plots of puncture position, angle, depth, and needle length, overlaid on a procedural schema. Furthermore, to account for potential within-patient clustering resulting from multiple puncture trajectories per patient, a patient-level sensitivity analysis (*n* = 15) was performed using the mean values of trajectory parameters for each individual patient.

The angular margin was calculated as the difference between the theoretical allowable angle and the measured puncture angle for each trajectory, and is presented as the mean angular margin below the theoretical limit with its standard deviation (SD).

## 3. Results

### 3.1. Positioning Feasibility and Patient Flow

A total of 16 consecutive patients were initially positioned on the dorsal-space mat for a planned posterior approach in the supine position. Successful positioning and trajectory establishment were achieved in 15 of 16 patients, yielding a positioning feasibility rate of 93.8% (15/16).

One of the 16 patients was unable to undergo the posterior approach despite being able to lie supine on the dorsal-space mat. This patient, a man in his 60s scheduled for CT-guided biopsy of a right lower-lobe lung mass, reported severe bilateral shoulder pain during the preprocedural interview and stated that prone and lateral positioning were intolerable. Based on this self-reported positional intolerance, the dorsal-space mat was selected. Although he could maintain a stable supine position on the mat, he was completely unable to elevate his right arm due to shoulder pain, resulting in insufficient dorsal working space for the posterior approach. Despite shoulder discomfort, lateral decubitus positioning was tolerable during trial, and the procedure was therefore converted on the table to a lateral approach. This case met the predefined exclusion criterion and was excluded from the assessment of technical success, procedural success, complications, and trajectory analysis.

### 3.2. Patient, Lesion, and Procedural Characteristics

Consequently, 15 patients were included in the final analysis cohort. Patient demographics, lesion characteristics, and procedural details are summarized in Table 1. Overall, the mean age was 76.2 ± 6.7 years, and the cohort comprised 12 males and 3 females. Documented clinical reasons for which the supine position was clinically required or preferred by operators included severe pain (*n* = 5), abdominal wall tenderness (*n* = 1), obesity (*n* = 2), anatomical changes in the anterior wall due to gastric conduit reconstruction (*n* = 1), the placement of the implantable cardioverter-defibrillator (ICD) pad required for sensing-off (*n* = 1), the need to maintain a stable position during concurrent endovascular procedures (*n* = 1), and the operator’s clinical judgment based on preprocedural CT that the supine posterior approach would provide a safer or more stable trajectory than unstable partial positioning (*n* = 4). Notably, no patients in this analyzed cohort had acute respiratory insufficiency or were mechanically ventilated. Detailed lesion characteristics and inter-group comparisons between the two mat height groups (15 cm vs. 20 cm) are summarized in Table 2. Across 15 patients, a total of 35 puncture trajectories were evaluated. Target lesions included renal masses (*n* = 6), retroperitoneal masses (*n* = 4), lung masses (*n* = 3), a rib mass (*n* = 1), and an iliopsoas abscess (*n* = 1). The 15-cm mat group included 10 patients and the 20-cm mat group included 5 patients. At the patient level, significant between-group differences were observed in sex distribution (*p* = 0.0220) and lesion location (*p* = 0.0215). Skin-to-lesion depth was numerically shallower in the 20-cm group than in the 15-cm group, although this difference did not reach statistical significance at the patient level (3.1 ± 1.6 cm vs. 5.5 ± 3.1 cm, *p* = 0.0719). No significant differences were observed in age, body mass index, or maximum lesion diameter.

The overall median procedure time across all 15 patients was 26.0 min (range, 11.0–178.0 min). By procedure type, the median procedure time was 23.0 min (IQR, 18.2–26.0 min) for biopsy (*n* = 10), 19.0 min for drainage (*n* = 1), and 65.0 min (IQR, 60.5–84.5 min) for radiofrequency ablation (*n* = 3). In one patient who underwent biopsy during a transcatheter arterial embolization (TAE) procedure, the total combined procedure time on the mat was 178.0 min; this long duration serves as an indicator that the patient was able to maintain a comfortable and stable supine posture on the dorsal-space mat throughout an extended multi-step intervention.

**Figure 2:**
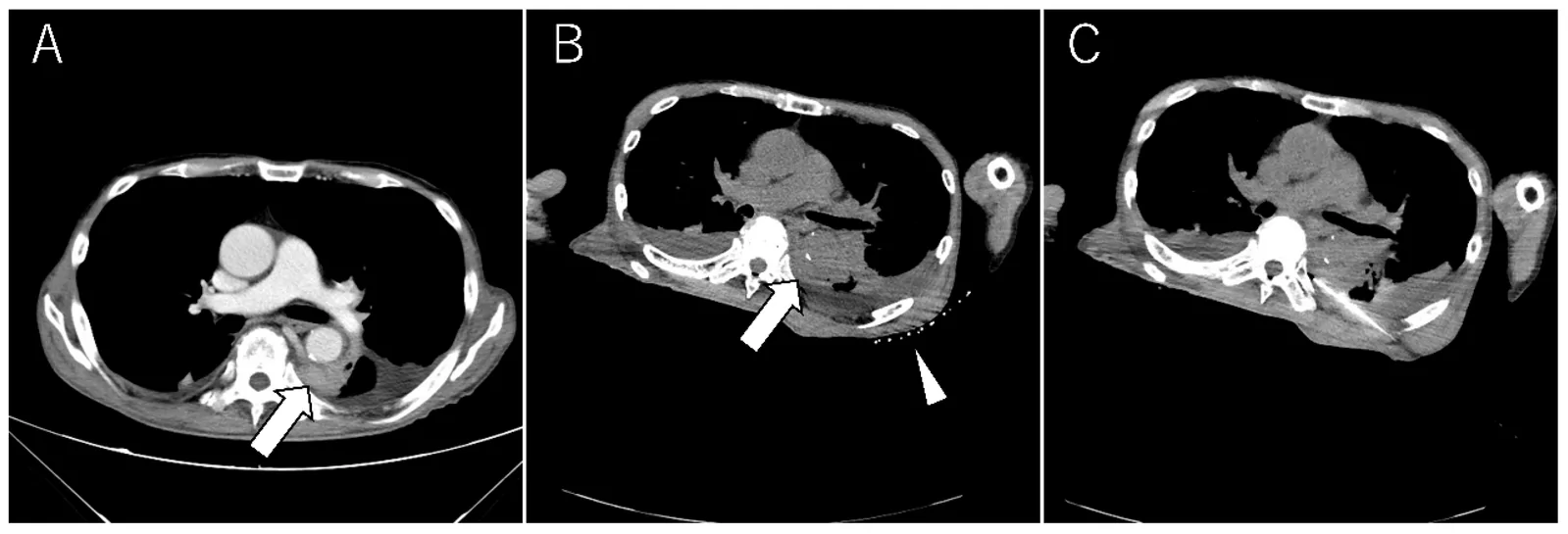
CT-guided lung biopsy using a dorsal-space mat with a posterior approach. (**A**) Preprocedural contrast-enhanced CT image. A soft-tissue mass (arrow) is seen posterior to the descending thoracic aorta, accompanied by pleural effusion. (**B**) Positioning CT image during the procedure. The patient is placed supine on the dorsal-space mat, with the puncture site marked on the back (arrowhead). The mat is highly radiolucent and therefore not clearly visible. (**C**) CT image during biopsy. Because the patient remains supine, the distribution of pleural effusion does not change, allowing biopsy along the planned puncture trajectory.

**Figure 3:**
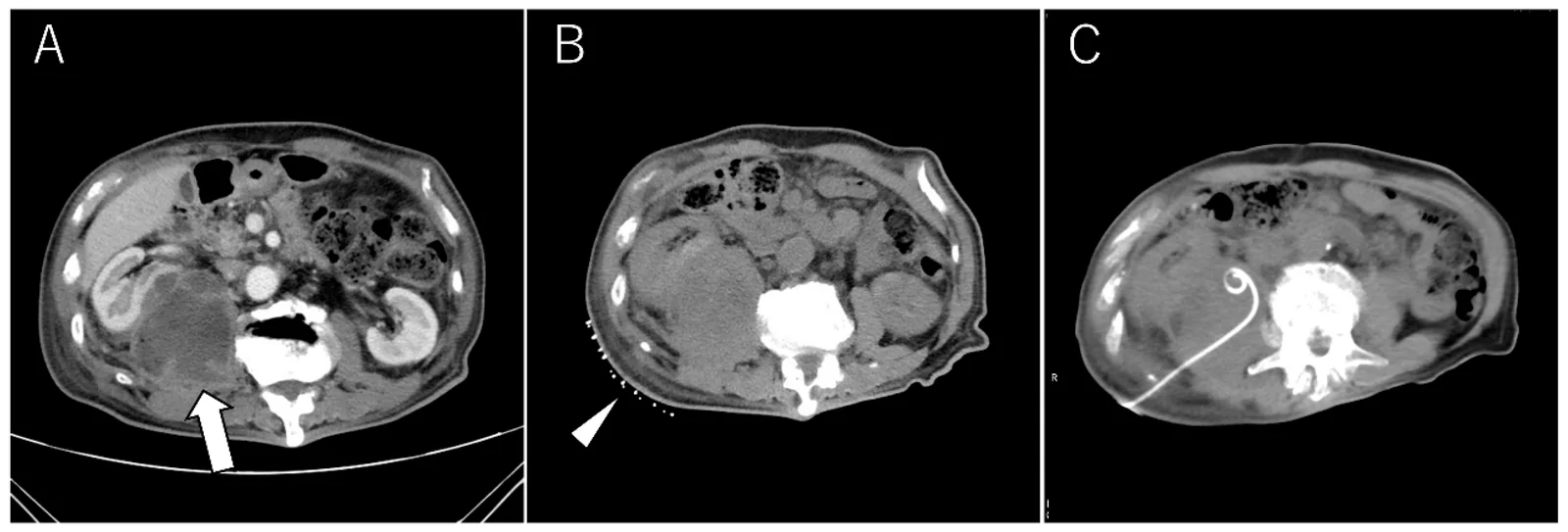
CT-guided drainage of a psoas abscess using a dorsal-space mat with a posterior approach. (**A**) Preprocedural contrast-enhanced CT image. A large abscess (arrow) is seen in the right psoas muscle. Due to severe back pain, drainage in the supine position was selected. (**B**) Positioning CT image during the procedure. The patient is placed supine on the dorsal-space mat, and the puncture site is marked (arrowhead). (**C**) CT image during drainage. The drainage tube was safely inserted via a posterior approach while the patient remained supine.

**Figure 4:**
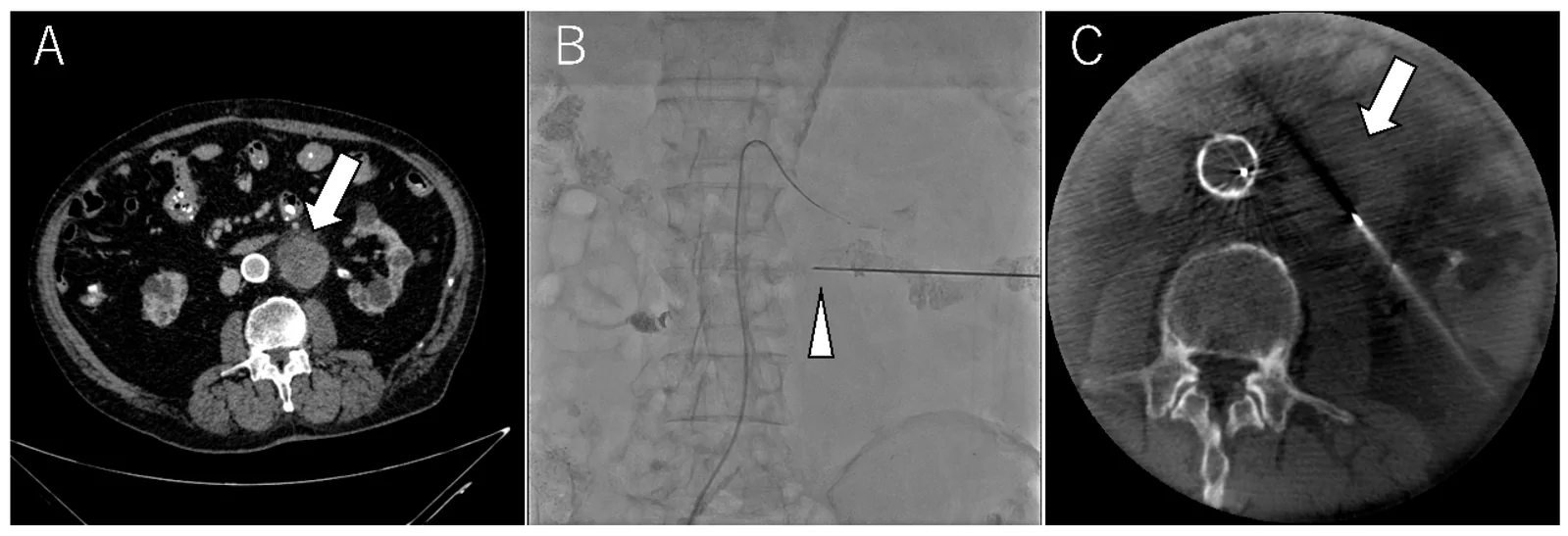
Cone-beam CT–guided biopsy of a retroperitoneal mass using a dorsal-space mat with a posterior approach. (**A**) Preprocedural contrast-enhanced CT image. A mass with surrounding inflammatory changes (arrow) is seen on the left side of the infrarenal abdominal aorta, raising suspicion for an infected thrombosed aneurysm. (**B**) Fluoroscopic spot image during puncture. Because biopsy was required to exclude malignancy, the procedure was performed in the angiography suite to allow rapid embolization if needed. Cone-beam CT–guided biopsy was performed using a posterior approach while the patient remained supine (arrowhead: biopsy needle). (**C**) Cone-beam CT image to confirm needle position. The biopsy needle reaches the mass (arrow) via a posterior approach. After advancing the needle an additional 1 cm, tissue sampling was performed under fluoroscopy.

Representative cases of CT-guided lung biopsy, CT-guided drainage of a psoas abscess, and CBCT-guided biopsy of a retroperitoneal mass are shown in Figure 2, Figure 3 and Figure 4.

### 3.3. Puncture Trajectory Analysis

Trajectory-related measurements for all puncture trajectories (*N* = 35) are summarized in Table 3. In the primary trajectory-level analysis, the horizontal puncture position (*x*_0_) was significantly smaller in the 20-cm mat height group compared to the 15-cm group (median [IQR]: 10.1 [2.9] cm vs. 12.9 [1.6] cm, *p* = 0.003). The puncture depth was also significantly shallower in the 20-cm mat height group (7.2 ± 2.3 cm vs. 9.3 ± 2.3 cm, *p* = 0.012). No significant differences were observed in vertical puncture position (*y*_0_, *p* = 0.234) or puncture angle (*p* = 0.740). Furthermore, to account for non-independence bias arising from multiple trajectories per patient (clustering effects), a patient-level sensitivity analysis (*n* = 15) was performed and is presented in Table 4. Although the direction of the between-group differences was similar to that observed in the primary trajectory-level analysis (*N* = 35), none of these differences remained statistically significant after the trajectories were collapsed to one mean value per patient.

**Figure 5:**
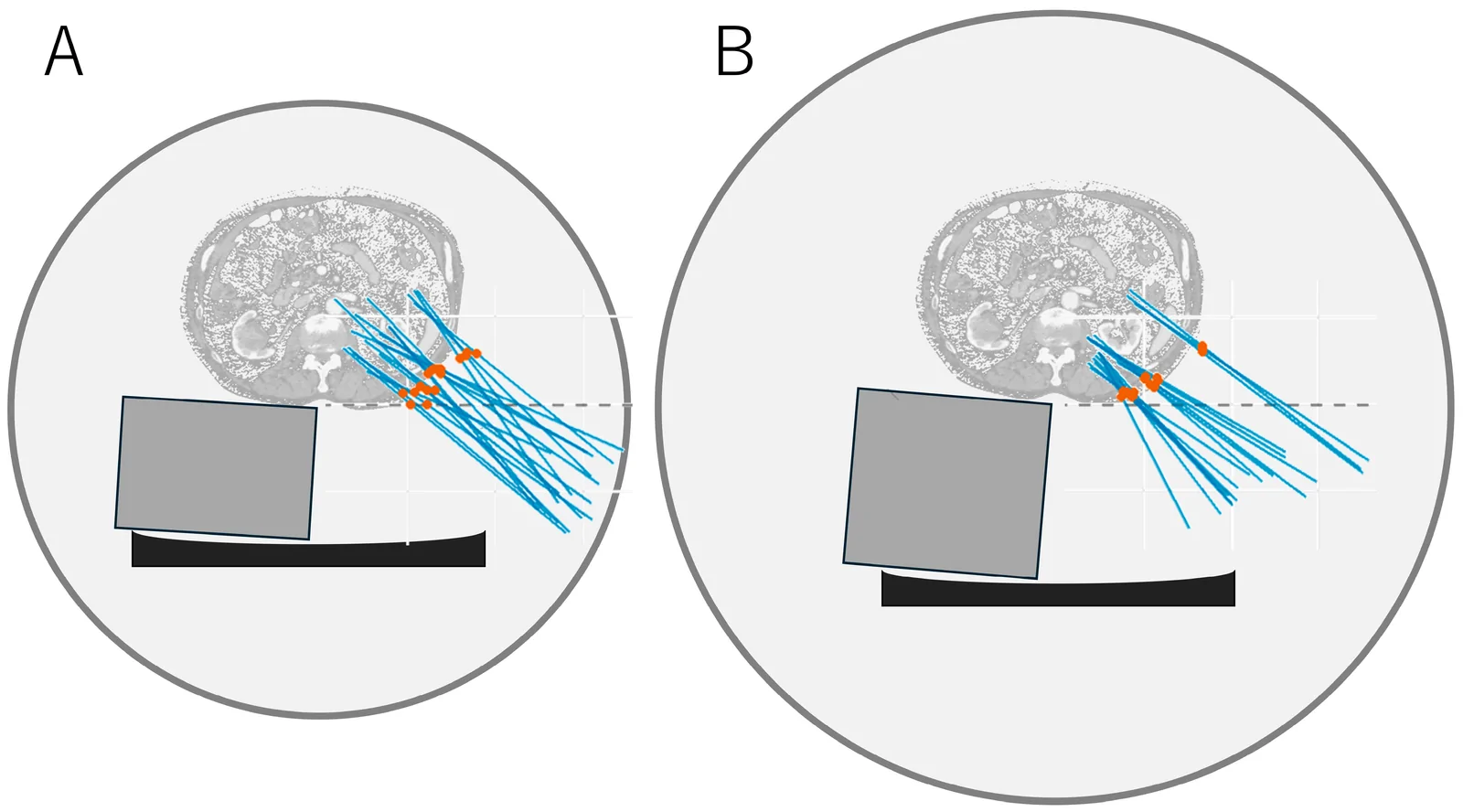
Geometric representation and spatial clearance of the dorsal-space approach within the CT gantry. The puncture trajectories reconstructed from actual patient data (*N* = 35) are overlaid on schematic illustrations integrating the CT table, tilted dorsal-space mats, and axial CT images framed within the CT gantry aperture. (**A**) Configuration combining a 70-cm gantry aperture diameter with a 15-cm mat height. (**B**) Configuration combining a 90-cm gantry aperture diameter with a 20-cm mat height. In each panel, the dashed line represents the horizontal reference line (y = 0), which, for geometric simplification, corresponds to the assumed flat dorsal body surface and the nominal upper-surface level of the dorsal-space mat. Orange points represent the actual entry points (*x*_0_, *y*_0_), and blue line segments delineate the individual needle trajectories, extending from the outer needle hub (needle length) to the target depth within the patient. The relationship between the gantry clearance and mat height demonstrates the physical feasibility and spatial margin of the upward puncture technique. The mat is shown tilted relative to the CT table to reflect the clinical setup. Axes and grid lines are intentionally retained to illustrate spatial scale and orientation.

**Figure 6:**
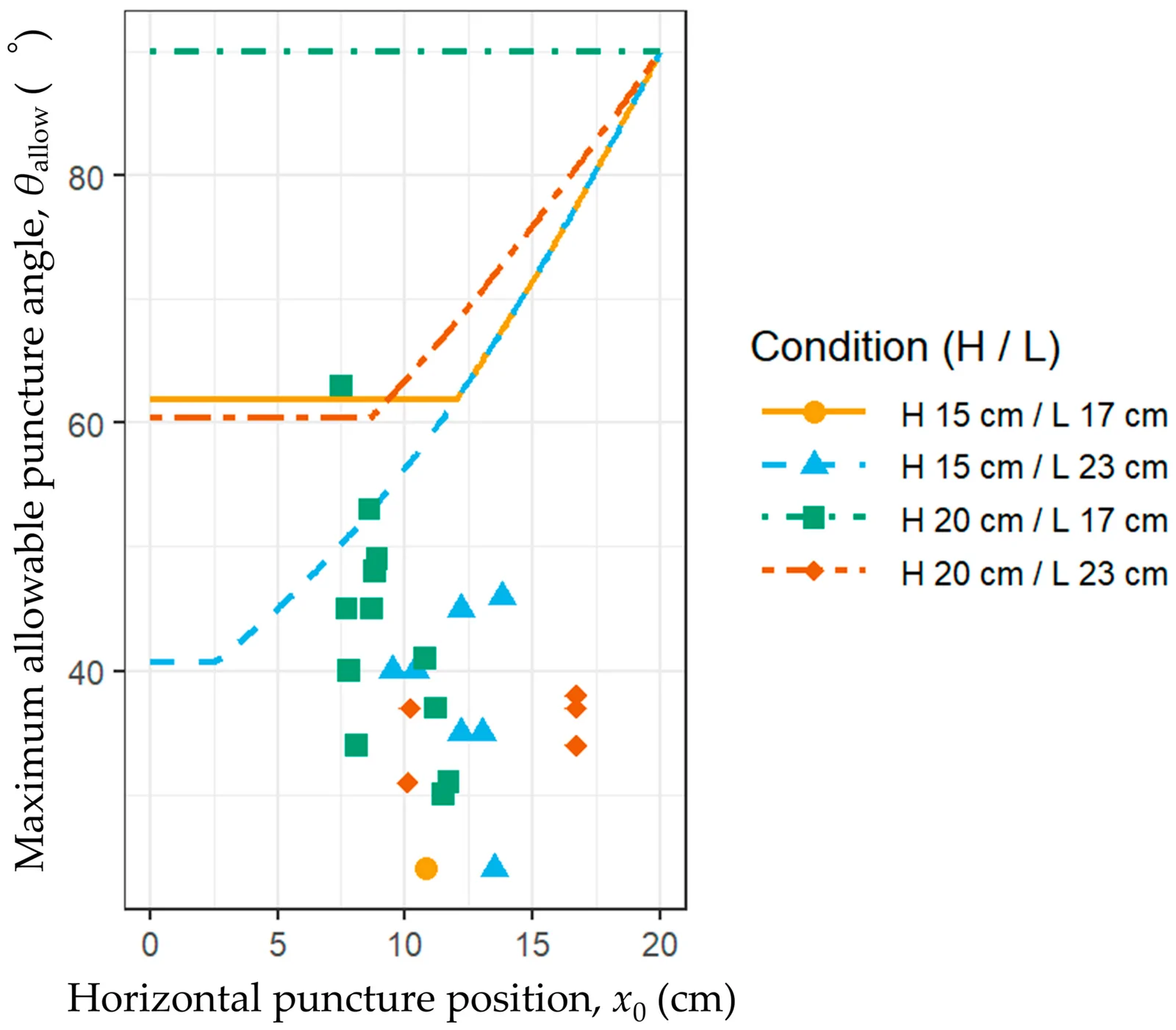
Comparison of theoretical maximum allowable puncture angles and actual clinical entry points. Solid and dashed lines represent the theoretical upper boundary of the allowable puncture angle (*θ*) as a function of horizontal puncture position (*x*_0_) across four combination conditions of mat height (*H* = 15 or 20 cm) and needle length (*L* = 17 or 23 cm). Scatter points indicate actual patient clinical data (*N* = 35). Needles with lengths of 20 cm and 22 cm are categorized under the 23-cm (*L* = 23 cm) group for theoretical comparison.

The spatial distribution of puncture trajectories for both mat heights is depicted in Figure 5. Furthermore, as shown in Figure 6, all measured puncture angles fell within the feasible geometric range predicted by the dorsal-space mat model, with a mean angular margin of 14.9° ± 10.9° below the theoretical limit.

### 3.4. Procedure Outcomes and Complications

Quantitative assessment of positional stability was feasible in 14 of the 15 successfully positioned patients. In the remaining CBCT-guided case, the dorsal-space mat was outside the field of view and could not be used as a reference for quantitative measurement. The median absolute horizontal and vertical displacements of the spinal canal center relative to the mat were 0.35 cm (IQR, 0.13–0.44 cm) and 0.19 cm (IQR, 0.05–0.42 cm), respectively. The median two-dimensional displacement was 0.41 cm (IQR, 0.33–0.45 cm). The median absolute change in axial rotation was 0.9° (IQR, 0.3–1.2°).

Technical success of needle placement into the target lesion was achieved in all cases (15/15, 100%). When evaluated per individual puncture trajectory (the primary analysis unit for geometric modeling), technical success was also achieved in all 35 trajectories (35/35, 100%). All procedures in these 15 patients were completed while maintaining a stable supine position on the dorsal-space mat.

Procedural success was achieved in all 15 cases. Adverse events, including intra-procedural and post-procedural events graded according to the SIR classification, are summarized in Table 5.

One postprocedural Grade 5 adverse event occurred after RFA for a renal tumor. Technical success was confirmed during the procedure on intraprocedural CT, and procedural success was confirmed on discharge CT performed one week later. However, shortly after discharge, the patient was readmitted with a urinary tract infection, subsequently developed pneumonia, and ultimately died of hemorrhagic shock caused by sudden upper gastrointestinal bleeding approximately two weeks after the procedure. Although a causal relationship with RFA could not be excluded, the clinical course indicated that the event was unrelated to the dorsal-space mat or the puncture trajectory.

## 4. Discussion

In this study, we conducted a preliminary feasibility evaluation of a supine posterior CT-guided approach using a dorsal-space mat in patients who were unable to tolerate the prone position for various procedures, including biopsy, drainage, and radiofrequency ablation (RFA). Given the small and heterogeneous cohort, the findings should be interpreted as preliminary and exploratory. Technical success was achieved in all cases, and each procedure type was completed as intended. One Grade 5 adverse event occurred following RFA. According to the CIRSE guidelines, major complications associated with RFA include infection (<1%), pneumothorax (<2%), and gastrointestinal perforation (1%) [16]. In the present case, urinary tract infection was considered potentially related to the ablation procedure, whereas pneumonia and gastrointestinal bleeding are not listed among typical RFA-related complications and their causal relationship with the intervention remained uncertain. Importantly, none of these events showed any direct association with the use of the dorsal-space mat or the posterior approach. Therefore, the adverse events observed in this study are unlikely to represent risks inherent to the dorsal-space mat itself. Nevertheless, given the limited sample size, further investigation is required to more definitively assess the safety profile of this technique.

When target lesions are located posteriorly, CT-guided interventions typically require a posterior approach, which in most cases necessitates prone positioning. However, prone or lateral positioning may be difficult or inappropriate in patients with severe pain, respiratory insufficiency, obesity, or those undergoing mechanically assisted ventilation under conscious sedation. In such situations, patients must remain supine, and CT-guided procedures may need to be abandoned or may be attempted but ultimately aborted due to inability to maintain the required position or the absence of a safe puncture window. Indeed, a large cohort study reported that 1.7% of CT-guided interventions were canceled or aborted for these reasons [8]. In our cohort as well, pain-related limitations and obesity were common reasons for inability to tolerate the prone position. Although sedated or mechanically ventilated patients were not included in this cohort, prone positioning may compromise respiratory management in such individuals. The dorsal-space mat may therefore be particularly useful when dorsal fluid collections or abscesses require intervention in patients who cannot safely tolerate prone positioning. Future prospective evaluation in this population is warranted. These patterns indicate that patients with pain-related limitations, altered anterior anatomy, or device-related constraints may particularly benefit from a supine posterior approach.

For patients restricted to the supine position, a posterior approach assisted by a dorsal-space mat has the potential to enable CT-guided procedures that were previously difficult or not feasible. Prior attempts to perform a posterior approach in supine patients have relied on wedges, cushions, or axial puncture techniques [9,10,11,12], but these methods have been limited by poor reproducibility and substantial operator dependence. Our study demonstrates that the dorsal-space mat can be systematically applied across multiple anatomical targets and procedure types, thereby expanding upon earlier evidence that had been limited to a single case report [13]. Nevertheless, because this study reflects early institutional experience, further investigations are required to validate the safety and efficacy of this technique. In addition to the supine posterior approach using the dorsal-space mat, supported lateral decubitus or semi-prone positioning are commonly used alternatives for accessing posterior targets. However, the relative burden of these positions varies among patients, and pain or insufficient stability often limited their feasibility in this cohort. Although the dorsal-space mat elevates the patient and requires attention to fall prevention during positioning and needle manipulation, it provided stable support and enabled a posterior route in patients who could not tolerate prone or lateral positioning.

Although the supine posterior approach was originally devised for patients who cannot tolerate the prone position, it may offer additional advantages. First, in adrenal lesions, prone positioning increases aeration of the non-dependent posterior lung base, making posterior needle advancement more difficult [17]. In contrast, the supine posterior approach reduces diaphragmatic motion and basal lung aeration, thereby facilitating needle placement near the diaphragm and potentially lowering the risk of pneumothorax. Second, body positioning alters not only the lungs but also the kidneys, bowel, and major vessels; thus, switching to the prone position may require modification of the needle path planned on diagnostic CT. Conversely, the supine posterior approach allows the procedure to be performed while preserving the anatomical relationships observed on diagnostic CT. These differences highlight the added value of the supine posterior approach compared with the conventional prone technique.

In the trajectory-level analysis, the 20-cm mat group showed a smaller horizontal puncture position (*x*_0_) and shallower puncture depth than the 15-cm mat group. However, neither difference remained statistically significant in the patient-level sensitivity analysis. Moreover, the two mat-height groups differed in several baseline and procedural characteristics. At the patient level, sex distribution and lesion location differed significantly between groups, while at the trajectory level, significant differences were also observed in lesion location, skin-to-lesion depth, and procedure type. Given these between-group imbalances, the observed differences in puncture position and depth should be interpreted as descriptive findings rather than as effects attributable to mat height.

Quantitative analysis of puncture position, angle, and depth revealed the distribution of trajectories achievable with the dorsal-space mat. This confirmed that the mat reliably creates sufficient posterior working space. Across all 35 trajectories, the proposed method reliably enabled an upward puncture at a mean angle of approximately 40°. Geometric analysis demonstrated that the maximum allowable puncture angle is governed by two factors: the geometric relationship between needle length and mat height near the midline, and the progressive release of angular constraints as the puncture site shifts laterally and clears the CT table edge. Theoretically, a greater mat height increases the available clearance between the patient and the CT table, thereby expanding the range of geometrically feasible trajectories and, in selected configurations, allowing steeper puncture angles from more medial entry points. All 35 puncture trajectories in this study fell within these theoretical limits, confirming that all observed trajectories were geometrically feasible within the theoretical constraints and supporting the potential utility of the geometric model for preprocedural planning. It should be emphasized that this geometric model is designed to define the theoretical ceiling (allowable upper limit) permitted by mat height, needle length, and table geometry, rather than to calculate a single “optimal” puncture angle. In clinical practice, the optimal puncture angle for an individual patient is inherently determined by the target lesion’s depth, local anatomy, and the presence of intervening structures. The observed mean angular margin of 14.9° ± 10.9° indicates that the measured trajectories remained below the theoretical geometric boundary without approaching its absolute limit. Operators should recognize that feasible puncture angles and positions are inherently restricted by CT gantry diameter, mat height, and the CT table edge. In our geometric model, the dorsal body surface was simplified as a flat, non-deformable plane. In clinical practice, two opposing factors affect the actual working space. On one hand, the natural upward curvature of the lateral body contour (+*x*, +*y* directions) provides extra spatial clearance, making our model’s theoretical calculations conservative. On the other hand, in obese patients, gravity can cause soft tissue to sag into the dorsal space provided by the mats, effectively narrowing the working space and reducing the maximum allowable puncture angle. To account for these dynamic anatomical variations, operators routinely evaluate the actual tissue contour and available space on preprocedural CT planning images before needle placement.

Maintaining the supine position throughout planning and needle placement may offer two distinct advantages. First, it avoids the anatomical changes associated with repositioning from the supine position to the prone, lateral, or oblique position. Clinically relevant changes in the relative positions of organs and other anatomical structures have been reported with changes in patient position [18,19], potentially requiring modification of a puncture route planned on preprocedural supine CT images or increasing the risk of unintended injury. Second, maintaining positional stability during the procedure is important for preserving a safe puncture window. Previous studies have reported that inability to maintain the required position may result in loss of a safe puncture window and consequent abortion of CT-guided interventions [8]. In the present study, quantitative comparison of planning and confirmation CT images showed a median two-dimensional displacement of 4.1 mm and a median absolute axial rotation of 0.9° relative to the dorsal-space mat. These findings quantitatively characterize the degree of positional change on the dorsal-space mat during the procedure.

Previous studies have identified non-prone positioning, including supine and lateral positions, as a risk factor for pneumothorax requiring chest drainage after CT-guided lung biopsy [2,3,4,5]. However, the relationship between patient position and pneumothorax risk may also depend on the direction of the puncture approach. A recent study demonstrated that an access route beginning in a dependent lung region was independently associated with a lower risk of pneumothorax, irrespective of patient position [20]. In addition, multiple studies have reported that placing the puncture site in the dependent position immediately after lung biopsy reduces the incidence of pneumothorax [21,22,23,24]. These findings suggest that the relationship between the access route and the dependent lung, rather than patient position alone, may be relevant to pneumothorax risk. The dorsal-space mat may provide a means of creating working space on the dependent side of the patient, potentially allowing a dependent access route to be selected according to lesion location. In the present study, this concept was applied to a posterior approach in the supine position; theoretically, the same principle could be applied to other patient positions, including an anterior approach in the prone position. Whether such positioning strategies reduce pneumothorax risk requires prospective evaluation.

The trajectory analysis also demonstrated that the dorsal-space mat provides sufficient working space for needle manipulation. The mean puncture angle of 40.2° and mean depth of 8.3 cm were well within the typical range of CT-guided procedures, and needle deviation could be readily controlled with forceps. Aside from advancing the needle in an upward direction, the procedure did not differ substantially from conventional CT-guided interventions and required no specialized techniques or devices. The method was compatible with both CT and CBCT systems, supporting its practicality across different imaging platforms.

This study has several limitations. First, it was retrospective with a limited sample size and therefore subject to selection bias. Because the technique was in an early phase of adoption, cases with relatively large or accessible lesions were more likely to be selected. Although positional stability was quantitatively assessed by comparing the relative position and axial rotation of the spinal canal between planning and confirmation CT images, this retrospective analysis was limited to axial positional changes and could not assess craniocaudal displacement or continuous intraprocedural motion. Furthermore, quantitative assessment was unavailable in the CBCT-guided case because the dorsal-space mat was outside the field of view. Because trajectory measurements were obtained only from procedures that had already been deemed feasible during preprocedural assessment, these measurements describe the output of successful puncture trajectories rather than directly testing procedural feasibility or stability. As a result, they cannot determine whether the technique would be feasible in patients with more severe positional intolerance. Second, the study reflects early experience from two institutions, and differences in operator preference or CT equipment may have influenced the results. Third, multiple puncture trajectories within the same patient were analyzed individually, and these observations were not independent. Because several trajectories originated from the same patient context, feasibility and safety outcomes could potentially be correlated. To evaluate the impact of within-patient clustering, we conducted a patient-level sensitivity analysis (*n* = 15). Although the direction of the between-group differences was similar to that observed in the trajectory-level analysis, none remained statistically significant after the trajectories were collapsed to one mean value per patient. This finding further underscores the exploratory nature of the between-group comparisons in this small cohort.

Fourth, the geometric model assumes a rigid, flat dorsal surface without tissue deformation. It does not account for soft-tissue sagging due to gravity in obese patients, which may restrict the working space compared with the theoretical model.

Fifth, all geometric trajectory measurements were performed by one of two operators (Y.K. or F.S.), with cases 1–10 measured by Y.K. and cases 11–15 measured by F.S. All measurements followed a unified measurement protocol using standard workstation tools. Because each case was measured by a single operator, inter-observer variability could not be assessed in this study. Future multi-reader studies should evaluate the reproducibility of these measurements. Sixth, although the dorsal-space mat improves the reproducibility of obtaining a feasible puncture trajectory, all procedures in this feasibility study were performed by highly experienced, board-certified interventional radiologists. Operator expertise may therefore have influenced procedural success, needle manipulation, and trajectory selection, and broader validation—including evaluation by less experienced operators—is needed to determine the generalizability of this technique. In addition, one patient died of gastrointestinal bleeding 2 weeks after the procedure. Although the event was considered unrelated to the dorsal-space mat or puncture trajectory, the small sample size precludes any definitive assessment of the safety of this technique. Larger studies are therefore required to further evaluate its safety.

Overall, this study should be regarded as a preliminary feasibility investigation. To more robustly evaluate this positioning method, future prospective studies should enroll all patients in whom prone positioning is not possible, documenting the proportion who can be positioned on the dorsal-space mat, the proportion requiring conversion to alternative approaches, and procedural outcomes in each group. In addition, future prospective work should incorporate three-dimensional or continuous measures of patient motion, differences between planned and achieved needle trajectories, and the number of needle adjustments or forceps-assisted stabilizations—as well as comparisons with the conventional prone approach.

## 5. Conclusions

The dorsal-space mat enables a posterior approach while maintaining the supine position and provides working space for CT-guided puncture across a variety of target lesions and procedure types. All observed puncture trajectories were geometrically feasible within the theoretical constraints of the model. These findings represent preliminary feasibility data, and further studies are needed to clarify the broader clinical role of this positioning method in patients for whom the supine position is required or preferred.

## Figures and Tables

**Table 1:** Overall Patient, Lesion, and Procedural Characteristics (*n* = 15 Patients, *N* = 35 Trajectories).

Parameter	Overall
Patient-level	(*n* = 15)
Age (years)	76.2 ± 6.7
Sex (Male/Female)	12/3
BMI (kg/m^2^)	23.7 ± 5.3
Reason for supine position	Spinal or joint pain (*n* = 5); abdominal wall tenderness due to chronic inflammation (*n* = 1); obesity (*n* = 2); others (*n* = 7)
Lesion location	
Renal tumor/mass	6
Lung mass	3
Retroperitoneal mass	4
Rib mass	1
Psoas abscess	1
Lesion long axis (cm)	5.1 ± 2.3
Lesion short axis (cm)	3.5 ± 1.6
Lesion depth (cm)	4.7 ± 2.9
Procedure type	
Biopsy	10
RFA	3
Biopsy following TAE	1
Drainage	1
Mat height used	15 cm (*n* = 10), 20 cm (*n* = 5)
Needle length used	17 cm (*n* = 4), 20 cm (*n* = 3), 22 cm (*n* = 1), 23 cm (*n* = 7)
Number of puncture trajectories per patient, median (range)	2 (1–4)
Trajectory-level	(*N* = 35)
Lesion location	
Renal tumor/mass	20
Lung mass	8
Retroperitoneal mass	5
Rib mass	1
Psoas abscess	1
Lesion long axis (cm)	4.6 ± 2.1
Lesion short axis (cm)	3.3 ± 1.5
Lesion depth (cm)	4.4 ± 2.3
Procedure type	
Biopsy	24
RFA	9
Biopsy following TAE	1
Drainage	1
Mat height used	15 cm (*n* = 18), 20 cm (*n* = 17)
Needle length used	17 cm (*n* = 15), 20 cm (*n* = 9), 22 cm (*n* = 1), 23 cm (*n* = 10)

Continuous variables are presented as mean ± standard deviation, median (range), or count (*n*). *n*, number of patients; *N*, number of trajectories; BMI, body mass index; RFA, radiofrequency ablation; TAE, transcatheter arterial embolization.

**Table 2:** Comparison of Baseline and Trajectory Characteristics Between 15-cm and 20-cm Mat Height Groups at Patient and Trajectory Levels.

Parameter	Overall	15-cm Mat Group	20-cm Mat Group	*p*-Value	
Patient-level	(*n* = 15)	(*n* = 10)	(*n* = 5)		
Age (years)	76.2 ± 6.7	75.0 ± 7.8	78.6 ± 3.4	0.2361	*
Sex (Male/Female)	12/3	10/0	2/3	**0.0220**	†
BMI (kg/m^2^)	23.7 ± 5.3	24.6 ± 5.1	22.0 ± 5.4	0.3891	*
Lesion location				**0.0215**	†
Renal tumor/mass	6	3	3		
Lung mass	3	1	2		
Retroperitoneal mass	4	4	0		
Rib mass	1	1	0		
Psoas abscess	1	1	0		
Lesion long axis (cm)	5.1 ± 2.3	5.5 ± 2.2	4.2 ± 2.5	0.3536	*
Lesion short axis (cm)	3.5 ± 1.6	3.6 ± 1.4	3.2 ± 2.0	0.6795	*
Lesion depth (cm)	4.7 ± 2.9	5.5 ± 3.1	3.1 ± 1.6	0.0719	*
Procedure type				0.4013	†
Biopsy	10	5	5		
RFA	3	3	0		
Biopsy following TAE	1	1	0		
Drainage	1	1	0		
Needle length (cm)	20.3 ± 2.7	20.8 ± 2.4	19.4 ± 3.3	0.4285	*
Trajectory-level	(*N* = 35)	(*N* = 18)	(*N* = 17)		
Lesion location				**0.0005**	†
Renal tumor/mass	20	9	11		
Lung mass	8	2	6		
Retroperitoneal mass	5	5	0		
Rib mass	1	1	0		
Psoas abscess	1	1	0		
Lesion long axis (cm)	4.6 ± 2.1	4.9 ± 1.9	4.3 ± 2.3	0.3591	*
Lesion short axis (cm)	3.3 ± 1.5	3.4 ± 1.2	3.1 ± 1.8	0.4845	*
Lesion depth (cm)	4.4 ± 2.3	5.5 ± 2.5	3.3 ± 1.5	**0.0039**	*
Procedure type				**0.0005**	†
Biopsy	24	7	17		
RFA	9	9	0		
Biopsy following TAE	1	1	0		
Drainage	1	1	0		
Needle length (cm)	19.6 ± 2.6	20.4 ± 2.1	18.8 ± 2.8	0.0546	*

Values are expressed as mean ± standard deviation or counts. Bold *p*-values indicate statistical significance (*p* < 0.05). * Welch’s *t*-test for continuous variables. † Fisher’s exact test (simulated) for categorical variables. *n*, number of patients; *N*, number of trajectories; BMI, body mass index; RFA, radiofrequency ablation; TAE, transcatheter arterial embolization. Note: Trajectory-level analyses (*N* = 35) contain non-independent observations from 15 patients. Refer to Table 4 for the patient-level sensitivity analysis (*n* = 15).

**Table 3:** Comparison of Puncture Trajectory Parameters (*N* = 35).

Parameter	Overall (*N* = 35)	15-cm Mat Group (*n* = 18)	20-cm Mat Group (*n* = 17)	*p*-Value	Statistical Test
Horizontal position, *x*_0_ (cm) *	11.7 [3.9]	12.9 [1.6]	10.1 [2.9]	**0.003**	Wilcoxon rank-sum test
Vertical position, *y*_0_ (cm) *	2.7 [2.7]	3.6 [2.5]	2.0 [1.8]	0.234	Wilcoxon rank-sum test
Puncture angle (°)	40.2 ± 9.1	39.7 ± 9.6	40.8 ± 8.8	0.740	Welch’s *t*-test
Puncture depth (cm)	8.3 ± 2.5	9.3 ± 2.3	7.2 ± 2.3	**0.012**	Welch’s *t*-test

Data are presented as mean ± standard deviation for normally distributed variables (Puncture angle and Puncture depth) or median [interquartile range, IQR] for non-normally distributed variables (*: *x*_0_ and *y*_0_). *p*-values in bold indicate statistical significance (*p* < 0.05). Note: Trajectory-level analyses (*N* = 35) contain non-independent observations from 15 patients. Refer to Table 4 for the patient-level sensitivity analysis (*n* = 15).

**Table 4:** Patient-level Sensitivity Analysis of Puncture Trajectory Parameters (*n* = 15).

Parameter	Overall (*n* = 15)	15-cm Mat Group (*n* = 10)	20-cm Mat Group (*n* = 5)	*p*-Value	Statistical Test
Horizontal position, *x*_0_ (cm) *	11.7 [3.8]	12.5 [2.2]	10.9 [3.5]	0.389	Wilcoxon rank-sum test
Vertical position, *y*_0_ (cm) *	2.5 [2.1]	2.5 [1.7]	2.9 [2.1]	0.748	Wilcoxon rank-sum test
Puncture angle (°)	38.9 ± 7.0	38.4 ± 7.4	39.9 ± 6.8	0.711	Welch’s *t*-test
Puncture depth (cm)	8.7 ± 2.9	9.5 ± 2.8	7.0 ± 2.7	0.128	Welch’s *t*-test

Data are presented as median [interquartile range, IQR] for non-normally distributed variables (*: *x*_0_ and *y*_0_) or mean ± standard deviation for normally distributed variables (Puncture angle and Puncture depth). *p*-values were calculated using the Wilcoxon rank-sum test or Welch’s *t*-test as appropriate based on normality testing.

**Table 5:** Procedural Outcomes (*n* = 15).

Procedure	Category	Value	Patient No, Adverse Event (Grade)
Biopsy (*n* = 11)	Procedural success	11/11	
	Procedural time, min		
	Biopsy alone (*n* = 10)	23 [18.2–26.0]	
	Biopsy following TAE (*n* = 1)	178	
	Intra-procedural AE	4/11	P11: small hemothorax (G1); P12: perirenal hematoma (G1); P13: perirenal hematoma (G1), hematuria (G1); P14: perirenal hematoma (G1)
	Post-procedural AE	0/11	
RFA (*n* = 3)	Procedural success	3/3	
	Procedural time, min	65 [60.5–84.5]	
	Intra-procedural AE	3/3	P1: pain (G1); P2: pain (G1); P5: pain (G1)
	Post-procedural AE	1/3	P2: urinary tract infection (G2), pneumonia (G2), GI bleeding (G5 *)
Drainage (*n* = 1)	Procedural success	1/1	
	Procedural time, min	19	
	Intra-procedural AE	0/1	
	Post-procedural AE	0/1	

Data for procedure time are presented as median [interquartile range] or absolute values. Abbreviations: AE, adverse event; G, Society of Interventional Radiology (SIR) grade; GI, gastrointestinal; RFA, radiofrequency ablation; TAE, transcatheter arterial embolization. * The clinical course indicated that the post-discharge upper GI bleeding was unrelated to the dorsal-space mat or puncture trajectory.

## Data Availability

Data supporting the findings of this study are available from the corresponding author upon reasonable request. Due to privacy and ethical restrictions related to clinical patient information and CT imaging data, raw datasets cannot be publicly shared. Summary data are contained within the article.
